# Prolonged Diuretic Activity and Calcium-Sparing Effect of *Tropaeolum majus*: Evidence in the Prevention of Osteoporosis

**DOI:** 10.1155/2014/958291

**Published:** 2014-06-16

**Authors:** Lorena Neris Barboza, Thiago Bruno Lima Prando, Paulo Roberto Dalsenter, Francielly Mourão Gasparotto, Francielli Gasparotto, Ezilda Jacomassi, Valdinei de Oliveira Araújo, Emerson Luiz Botelho Lourenço, Arquimedes Gasparotto Junior

**Affiliations:** ^1^Departamento de Farmacologia, Universidade Federal do Paraná, P.O. Box 19031, 81531-990 Curitiba, PR, Brazil; ^2^Instituto de Ciências Biológicas, Médicas e da Saúde, Universidade Paranaense, P.O. Box 224, 87502-210 Umuarama, PR, Brazil; ^3^Laboratório de Farmacologia Cardiovascular, Faculdade de Ciências da Saúde, Universidade Federal da Grande Dourados, Rodovia Dourados, Itahum, km 12, P.O. Box 533, 79.804-970 Dourados, MS, Brazil; ^4^Laboratório de Fitopatologia, Centro Universitário Cesumar, P.O. Box 204, 87050-900 Maringá, PR, Brazil

## Abstract

Although several studies indicate high effectiveness in the use of the hydroethanolic extract from *Tropaeolum majus* (HETM) as a diuretic, the impact of its prolonged use in the presence of low estrogen levels remains unclear. Thus, the aim of this study was to investigate the diuretic effects of prolonged administration of HETM in ovariectomized rats and their interrelationship between calcium excretion and bone turnover. Forty-two female Wistar rats were ovariectomized (OVX) and treated orally with different doses of HETM (3, 30, and 300 mg/kg) for 4 weeks. On the first day of treatment and at weekly intervals for four weeks the diuretic activity was evaluated. Electrolyte concentrations and creatinine levels were estimated from urine sample of each rat. The serum lipids, urea, creatinine, and osteocalcin were also measured at the end of the experiment. The data revealed that the HETM was able to sustain its diuretic effect after prolonged treatment. Moreover, its use has not affected the urinary calcium or potassium excretion, reduces lipid levels, and maintains osteocalcin levels similarly to untreated rats. These findings support the potential of HETM as a candidate to be used in clinical conditions in which the renal loss of calcium is not desired.

## 1. Introduction

Menopause is a physiological process that is characterized by the end of the reproductive period and results in the failure of the endocrine activity of the ovaries, particularly their inability to produce estrogens. Usually it occurs between 45 and 54 years of age and can present a number of comorbidities (mainly bone loss and cardiovascular disease) influenced by lifestyle, age, and genetic characteristics [1].

Recent studies show a close relationship between the reduction of estrogen levels and development of osteoporosis, hypertension, and acute myocardial infarction [2]. Several etiologies have been identified as being responsible for these changes; however, currently it is believed that the low levels of calcium, vitamin D, and potassium or excessive intake of sodium may be involved [3]. Calcium is the most abundant mineral in the human body, and its deficiency has correlation to several heart diseases and to important bone loss [4].

About 50% of women after menopause may have hypertension, and as first-line treatment, diuretic drugs are employed. Despite their high efficacy, drugs such as furosemide, which is calcium depletory, can aggravate bone loss and abbreviate the onset of osteoporosis [5]. Furthermore, these drugs are associated with several other adverse effects such as acquired diabetes, dehydration, arrhythmias, and disturbances in sexual function [6].

Recently, we have showed the efficacy of the hydroethanolic extract obtained from* Tropaeolum majus* (HETM) leaves as a diuretic and an antihypertensive, relating its effects to the flavonoid isoquercitrin, which was able to inhibit the angiotensin-converting enzyme and release bradykinin and prostaglandins [7–10]. Furthermore, Gasparotto Jr. et al. [7] and Gomes et al. [11] reported absence of toxicity after acute and subchronic administration of HETM in rats. Likewise, HETM was unable to produce (anti)estrogenic or (anti)androgenic activities in the short-term* in vivo* screening assays performed [12].

Although several preclinical studies indicate high effectiveness and safety in the use of this natural product as a diuretic, the impact of its use on the levels of calcium in the presence of low estrogen levels remains unclear. Thus, the aim of this study was to investigate the diuretic effects of prolonged administration of HETM in ovariectomized rats and evaluate the interrelationship between calcium excretion and bone turnover.

## 2. Materials and Methods

### 2.1. Plant Material and Preparation of the Hydroethanolic Extract from *Tropaeolum majus* L. (HETM)

The leaves of* Tropaeolum majus* were collected at the botanical garden of Universidade Paranaense (UNIPAR) (Umuarama, Brazil), which is located at 430 m altitude above sea level (S23°47′55–W53°18′48). A voucher specimen is deposited at the Herbarium of this university under the number 2230. The leaves were air-dried in an oven at 40°C for 4 days and then cut and pulverized. The resulting dried powdered plant material was macerated for 7 days in 90% ethanol solution. The solvent was removed using a rotary vacuum evaporator under reduced pressure and lyophilized, producing the HETM equivalent to 14.99% of the dried plant material. The chemical composition of HETM has been previously described [9].

### 2.2. Pharmacological Studies

#### 2.2.1. Animals

Three-month-old female Wistar rats from the colony of the Federal University of Paraná (Brazil) were used. The animals were maintained with a constant 12 h light/dark cycle and controlled temperature (22 ± 2°C). Standard pellet food (Nuvital, Curitiba/PR, Brazil) and water were available* ad libitum*. All experimental procedures were previously approved by the Institutional Ethics Committee of the Universidade Paranaense (authorization no. 22833/2012).

#### 2.2.2. Ovariectomy

Forty-two female rats were anesthetized with ketamine (100 mg/kg) and xylazine (20 mg/kg) intraperitoneally. Ovariectomy was performed by bilateral incision in prepubic access for removal of the ovaries (OVX group) as previously described [13]. A group called SHAM-operated had their ovaries exposed and repositioned to simulate the surgical stress. All incised regions received simple interrupted suture with 4–0 silk threads. For the surgical prophylaxis it was administered benzyl penicillin and streptomycin at doses of 160.000 IU and 70 mg/kg (intramuscularly), respectively.

#### 2.2.3. Evaluation of Prolonged Diuretic Activity

One week after surgery the animals were divided into 6 groups (*n* = 7). The diuretic activity was determined according to a method previously described with minor modifications [7]. Different groups of OVX rats received different daily doses of the HETM (3, 30, and 300 mg/kg) or furosemide (C+: 25 mg/kg) for 4 weeks. The animals of the SHAM-operated and OVX-negative control group (C−) were treated with vehicle alone. On the first day of treatment (outset) and at weekly intervals for four weeks, the animals received an oral dose (5 mL/100 g) of saline (0.9% NaCl) to impose a uniform water and salt load. After this procedure, the animals were kept in metabolic cages and the urine was collected in a graduated cylinder, and its volume was recorded at 2 h intervals for 8 h. Cumulative urine excretion was calculated in relation to body weight and expressed as mL/100 g. Electrolyte concentrations (Na^+^, K^+^, Cl^−^, Ca^++^, and HCO_3_^−^), pH, density, conductivity, and creatinine levels were estimated from urine sample of each rat. The serum concentration of total cholesterol (TC) and fractions (HDL-C and LDL-C), triglycerides, urea, creatinine, and osteocalcin were also measured at the end of the experiment (4th week).

*2.2.3.1. Analytical Procedures.* For serum analysis, blood samples were collected in conical tubes after decapitation. Serum was obtained by centrifugation (800 g, 10 minutes, 4°C) and stored at −20°C until their analysis. Electrolyte concentrations were quantified by flame photometry (Na^+^and K^+^) or titration (Cl^−^ and HCO_3_^−^). The pH and conductivity were directly determined on fresh urine samples using a Q400MT pH meter and a Q795M2 conductivity meter (Quimis Instruments, Brazil), respectively. Density estimation was made by weighing with a Mettler AE163 (±0.1 mg) analytical balance. The urinary calcium, TC, HDL-C, LDL-C, triglycerides, urea, and creatinine were determined by colorimetric method by an automated analyzer BM/Hitachi 912 (Cobas Mira, Roche, Indianapolis, USA). Serum levels of osteocalcin were measured by electrochemiluminescence in an automated analyzer photomultiplier (PMT).

### 2.3. Statistical Analysis

The results were expressed as the mean ± standard error of the mean (SEM). Statistical analyses were performed using one- or two-way analysis of variance (ANOVA) followed by Bonferroni's multiple comparison test. A *P* value less than 0.05 was considered statistically significant. The graphs were plotted and statistical analyses were performed using GraphPad Prism version 5.0 for Mac OS X.

## 3. Results

### 3.1. HETM Induces Sustained Diuresis and Natriuresis after Prolonged Treatment in OVX Rats

**Figure 1:**
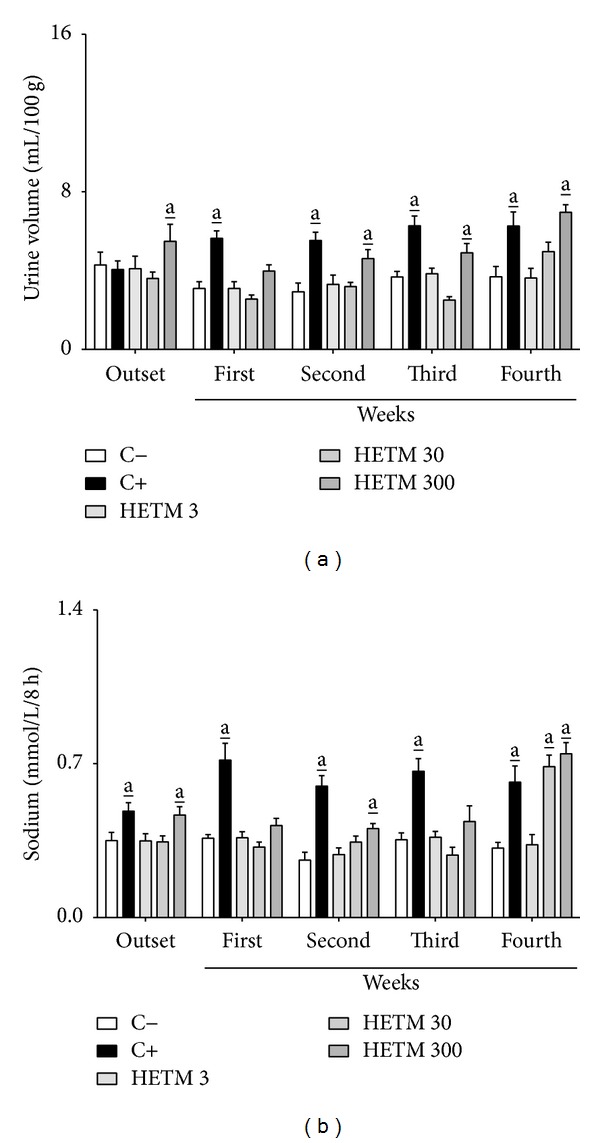
HETM induces sustained diuresis and natriuresis after prolonged treatment in OVX rats. Different groups of rats were treated for 4 weeks with HETM (3, 30, and 300 mg/kg), furosemide (C+, 25 mg/kg), or vehicle (C−). On the first day of treatment (outset) and at weekly intervals for four weeks, the urine was collected for 8 hours and its volume (a) and sodium concentration (b) were measured. Each bar represents the mean of 7 animals and the vertical lines show the S.E.M. $\underline{\text{a}}$ denotes significance levels compared with the group treated with vehicle alone (two-way ANOVA followed by Bonferroni test) ($\underline{\text{a}}$ *P* < 0.05).

The results for urine volume and urinary sodium excretion are shown in Figure 1. The HETM at the dose of 300 mg/kg was able to significantly increase the urine volume after acute administration (outset) and from the second week of treatment (Figure 1(a)). Urine volume in animals treated with furosemide was similar to those obtained after administration of HETM at its highest dose. Likewise, values for the excretion of sodium were significantly increased in the first administration and after the second and fourth weeks of the treatment with HETM at dose of 300 mg/kg (Figure 1(b)). Additionally, urine volume and urinary sodium excretion were not statistically different between SHAM-operated and OVX rats (data not shown). All other urinary parameters, including creatinine and potassium levels, were not affected by the treatment with HETM at all doses used (data not shown).

### 3.2. HETM Prevents the Elevation of Serum Cholesterol in OVX Rats

**Figure 2:**
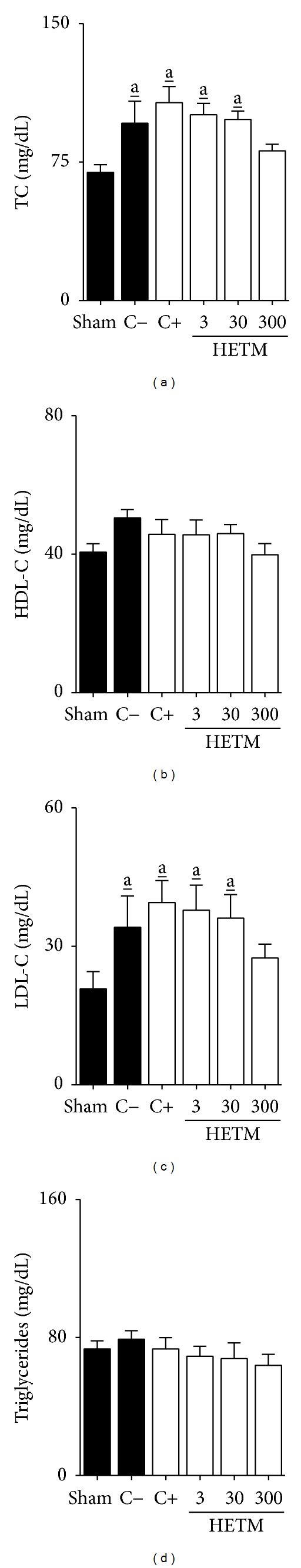
Prolonged HETM administration prevents the elevation of serum total cholesterol in OVX rats. Different groups of rats were treated for 4 weeks with HETM (3, 30, and 300 mg/kg), furosemide (C+, 25 mg/kg), or vehicle (C−). At the end of the experiment the animals were euthanized and their serum was collected for the determination of CT (a), HDL-C (b), LDL-C (c), and triglycerides (d). Each bar represents the mean of 7 animals and the vertical lines show the S.E.M. $\underline{\text{a}}$ denotes significance levels compared with the group treated with vehicle alone (one-way ANOVA followed by Bonferroni test) ($\underline{\text{a}}$ *P* < 0.05). TC: total cholesterol; HDL-C: high-density lipoprotein cholesterol; LDL-C: low-density lipoprotein cholesterol.

The results obtained for the levels of serum lipids (TC, HDL-C, LDL-C, and triglycerides) are shown in Figure 2. In all ovariectomized rats a significant increase in TC and LDL-C levels was observed, while HDL-C was maintained similarly to the SHAM-operated group. On the other hand, the treatment with HETM (300 mg/kg) was able to prevent the increase of TC and LDL-C levels in OVX rats, whereas furosemide did not show any effect on the levels of cholesterol or triglycerides (Figures 2(a)–2(d)). All other parameters (including urea and creatinine) showed no statistically significant differences between all experimental groups (data not shown).

### 3.3. Osteoprotective Effects of HETM in OVX Rats

**Figure 3:**
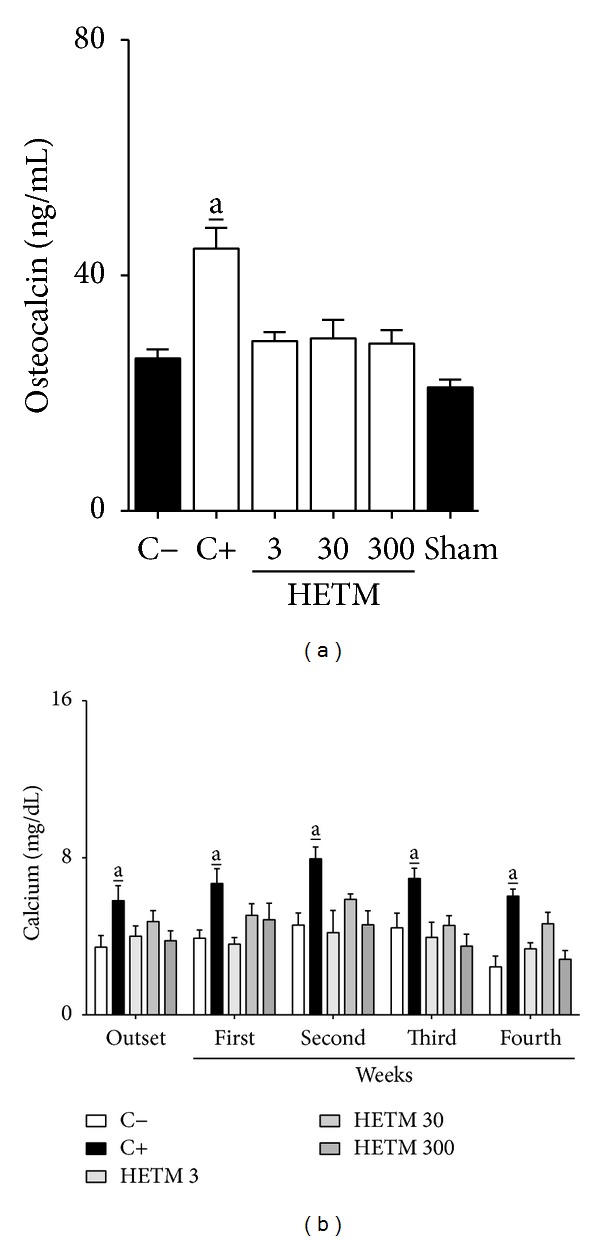
HETM induces calcium-sparing effects without affecting osteocalcin levels in OVX rats. Different groups of rats were treated for 4 weeks with HETM (3, 30, and 300 mg/kg), furosemide (C+, 25 mg/kg), or vehicle (C−). On the first day of treatment (outset) and at weekly intervals for four weeks the urine was collected and calcium concentration (b) was measured. At the end of the experiment the animals were euthanized and their serum was collected for the determination of serum osteocalcin (a). Each bar represents the mean of 7 animals and the vertical lines show the S.E.M. $\underline{\text{a}}$ denotes significance levels compared with the group treated with vehicle alone. One- (a) or two-way (b) ANOVA followed by Bonferroni test ($\underline{\text{a}}$ *P* < 0.05).

Serum levels of osteocalcin and urinary calcium in SHAM-operated and OVX rats treated for 4 weeks with HETM are shown in Figure 3. All OVX animals showed a high baseline level of osteocalcin compared to the SHAM-operated group. On the other hand, the animals of group C+ presented a significant increase in osteocalcin levels after four weeks of treatment (44.9 ± 3.55 ng/mL) when compared to the OVX-negative controls (25.8 ± 1.52 ng/mL) (Figure 3(a)). All animals treated with HETM (3, 30, and 300 mg/kg) maintained osteocalcin levels similar to those obtained in OVX rats treated with vehicle alone (C−). Likewise, while furosemide caused a significant renal excretion of calcium (approximately 50% higher than the control in all measurements), the HETM exhibited an important calcium-sparing effect (in all doses), similar to all groups treated with vehicle alone (Figure 3(b)). Renal excretions of calcium between the untreated controls (SHAM-operated and OVX rats) were not statistically different (data not shown).

## 4. Discussion

Menopause is characterized by depletion of ovarian follicles and results in a significant deficit in the production and release of estrogen. One common consequence of reduction or abolishment of this hormone in postmenopause period is the elevation of serum lipids and blood pressure, reduced bone mineral density, and, in some cases, osteoporosis [1]. Postmenopausal osteoporosis (experimental or natural) characterized by hypoestrogenism can directly influence the osteoblast proliferation and differentiation, leading to changes in levels of bone turnover markers [14].

Several markers of bone turnover, especially those synthesized by osteoblasts (as osteocalcin), are clinically used as predictors of postmenopausal osteoporosis. Unlike the other biochemical markers of bone metabolism, osteocalcin can rise in all conditions in which there is a renewal of bone structure, as occurs during childhood, after bone fractures, or osteoporosis induced by estrogen deficiency [15].

Osteocalcin, also known as bone gamma-carboxyglutamic acid-containing protein (BGLAP), is a noncollagenous protein found in bone and dentin, which plays an important role in bone mineralization. Osteocalcin is originated from osteoblastic synthesis and it is deposited into bone or released into circulation, where it correlates with histological measures of bone formation [16]. Additionally, osteocalcin may activate monocyte differentiation, resulting in an increased number of active bone-resorbing cells and promoting osteoclastic differentiation [17]. So, high levels of this protein, especially during the absence of estrogen, may indicate an increase in bone turnover with consequent changes in bone structure.

As the central role of osteocalcin is bone mineralization, the body calcium levels can profoundly affect its production and homeostasis. Calcium metabolism is very important during postmenopausal osteoporosis, and the intricate relationship between the reduction in estrogen levels and bone loss can be directly related to this ion [18]. Several external factors may be related to the acceleration of osteoporosis, including low calcium and vitamin D intake, insufficient sunlight exposure, and the use of some drugs that increase calcium excretion [5, 19]. Furosemide, a loop diuretic, is the largest depleting calcium among clinical diuretics and its prolonged use can negatively affect the development of osteoporosis [6]. Excessive calcium excretion, such as the furosemide-induced in this study, can affect the function of osteoblasts and therefore the production of various substances related to bone mineralization, including osteocalcin. Furthermore, it is known for a long time that loop diuretics may increase serum levels of CT and LDL-C and reduce levels of HDL-C [6]. If we consider that hyperlipidemia is present in postmenopausal women, the use of loop diuretics in these conditions can significantly increase the levels of serum lipids and lead to an increased cardiovascular risk of these patients.

The use of diuretics during menopause is quite widespread due to the high prevalence of hypertension in this population [2]. Moreover, many postmenopausal women use various natural products as diuretic without taking into account the impact of these compounds on serum lipids, calcium concentration, or bone metabolism. Our results bring evidence to light and show for the first time that HETM, despite its remarkable diuretic effect observed here and already previously reported in other experimental conditions [3, 4], was able to prevent the increase of serum cholesterol and excretion of calcium, maintaining levels of osteocalcin within normal range for the OVX rats. These effects may represent important aspects of safe use of this product as a natural diuretic, especially in a fast-growing population group in the world.

## 5. Conclusions

In conclusion, the results presented here demonstrate that HETM is able to sustain its diuretic activity after prolonged treatment in ovariectomized rats. Furthermore, we observed a significant natriuretic and potassium and calcium-sparing effect without adversely affecting serum lipids or osteocalcin levels. Although the flavonoid isoquercitrin was identified as being likely responsible for the diuretic effects of HETM [4], further studies are needed to evaluate its role as a protective agent in this experimental model. Taken together, these findings support the potential of a preparation derived from* Tropaeolum majus* as a candidate to be a phytomedicine used as a diuretic in patients with low levels of estrogen. Additional preclinical and clinical studies can clarify the role of this natural product in treating cardiovascular disorders associated with clinical conditions in which the renal loss of calcium is not desired.
